# Supplementary material for: Pseudogenization of the cntQ permease confers distinct yersinopine-metal uptake selectivity in Yersinia species
Source: Biometals. 2026 May 13;39(5):1583–93. doi: 10.1007/s10534-026-00816-8 (PMC13518427; doi:10.1007/s10534-026-00816-8)
Supplement: Supplementary file 1 [file 10534_2026_816_MOESM1_ESM.docx]

**Supplemental material:**

**CntP**

**CntQ**

**CntR**

**CntL**

**CntM**

**CntI**

**Supplementary Figure 1.** Sequence conservation between the different predicted cnt genes in *Y. pestis* and *Y. pseudotuberculosis*.

**Supplementary Figure 2.** AlphaFold3 models of a CntQ monomer from *Y. pseudotuberculosis* (YPTB1370; left) and *Y. pestis* (YP2838-40; right). The models are coloured according to the quality of the prediction, except for the right panel, which shows the position of the three peptides and the location of their N- and C-terminus. The approximate position of the membrane is shown in grey.

**Supplementary Figure 3.** AlphaFold3 models of the CntPQ_2_R_2_ from *Y. pseudotuberculosis* (left ; ipTM = 0.81 pTM = 0.84) and the CntQ_2_ from *Y. pestis* (right ; ipTM = 0.61 pTM = 0.61). Although there would be enough space for interaction with the ATPase CntR in the cytoplasm, the space used for recognition of CntP in the periplasm is occupied by additional peptide parts that have no counterpart in *Y.* *pseudotuberculosis*, therefore leading to our central hypothesis that the three peptides produced by Y. pestis (Y2838-40), even if produced, are likely non-functional.

**Supplementary Figure 4.** AlphaFold3 models of the CntPQ_2_R_2_ from *Y. pseudotuberculosis* (left; ipTM = 0.81 pTM = 0.84) and the equivalent from *Y. pestis* (right; ipTM = 0.65 pTM = 0.65). With regard to *Y. pestis*, the modelling with CntP indicates that the parts that have no equivalent in *Y. pseudotuberculosis* now folds in the membrane (arrows), most probably because the model forces CntP binding. However, if stable, this part should fold in the periplamic space, which should therefore prevent either membrane insertion of CntQ or CntP binding to CntQ, anyway likely impairing its function.

**Supplementary Figure 5**: SDS-PAGE gels illustrating the purifications of YpCntL (left) and YpCntM (right) using HisTrap columns. NR corresponds to the flow-through fraction, 1, 2 and 3 to the different eluted fractions, and P to the eluted fraction of CntM.

**Supplementary Figure 6.** Zur-dependent repression of the *cnt* operon in *Y. pestis* and *Y. pseudotuberculosis*. *Y.* *pestis* and *Y. pseudotuberculosis* Δ*lacZ* strains, either expressing (+) or lacking (*-*) Zur, carried a chromosomal fusion between the *cnt* operon promoter (P*_cntP_*) and the *E. coli* *lacZ* reporter gene. Promoter activity was assessed by β-galactosidase activity, visualized as blue coloration on LB agar plates supplemented with X-gal.

**Supplementary Figure 7:** Growth curves (A) and inferred growth rates (B) of *Y. pseudotuberculosis* cultivated in TMH without or with increasing concentration of EDTA. In A, the data represent the means and the confidence intervals (95%) calculated from three biological replicates. In B, the data from these three biological replicates are shown as individual points associated with their mean and standard deviation (in grey).

**Supplementary Table 1. Pseudogenization of the *cntQ* permease is conserved in *Y. pestis* strains.** List of orfs predicted in sequenced *Y. pseudotuberculosis* and *Y. pestis* strains at the *cntQ* position. maxLrap and minLrap denote respectively the length (expressed as a percentage of the gene) that is conserved, taking YPTB1370 as a reference (Query). BeginQ and EndQ refer to portions of sequence that is conserved on YPTB1370, taking YPTB1370 (Query) as a reference. BeginB and EndB refer to portions of sequence that is conserved in a given sequence (Bait), using YPTB1370 as a reference.

**Supplementary Table 2**. **Bacterial strains and plasmids used in this study**

| *Strain name in the text* | | *Description* | |
| --- | --- | --- | --- |
| ***Yersinia pseudotuberculosis*** | | | |
| *Y. pseudotuberculosis WT* | | *Y. pseudotuberculosis* IP32777 | |
| *Y. pseudotuberculosis* Δ*lacZ* P*_cntP_*::*lacZ* (Zur+) | | IP32777 Δ*lacZ* P*_cntP_*::*lacZ*  chromosomal *lacZ* reporter fusion with *cnt* promoter | |
| *Y. pseudotuberculosis* Δ*lacZ* P*_cntP_*::*lacZ* (*Z*ur-) | | IP32777 Δ*lacZ* Δ*zur* P*_cntP_*::*lacZ*  *zur* deletion and *lacZ* reporter fusion with *cnt* promoter | |
| *Y. pseudotuberculosis ΔcntLMI* | | IP32777 Δ*cntLMI*-FRT  unlabeled deletion of *cntLMI* | |
| *Y. pseudotuberculosis ΔcntQ(RLMI)* | | IP32777 Δ*cntQ*::Km1447  *cntQ* deletion and insertion of a kanamycin resistance cassette abolishing *cntRLMI* expression | |
| *Y. pseudotuberculosis ΔcntQ* | | IP32777 Δ*cntQ*-FRT  unlabeled deletion of *cntQ,* no polar effect on *cntRLMI* downstream genes | |
| *Y. pseudotuberculosis ΔcntQ(RLMI)* complemented | | IP32777 Δ*cntQ*::Km1447 mini-Tn7-Zeo2::*cntPQRLMI*  Strain complemented by the insertion of *cntPQRLMI* | |
| ***Yersinia pestis*** | | | |
| *Y. pestis WT* | | *Y. pestis* KIM6+ | |
| *Y. pestis ΔcntLMI* | | KIM6+ Δ*cntLMI*::Tp1087  *cntLMI* deletion and insertion of a trimethoprim (Tp) resistance cassette | |
| *Y. pestis ΔcntQ(RLMI)* | | KIM6+ Δ*cntQ*::Km1447  *cntQ* deletion and insertion of a kanamycin resistance cassette abolishing *cntRLMI* expression | |
| *Y. pestis* Δ*lacZ* P*_cntP_*::*lacZ* (Zur+) | | KIM6+ Δ*lacZ* P*_cntP_*::*lacZ*  chromosomal *lacZ* reporter fusion with *cnt* promoter | |
| *Y. pestis* Δ*lacZ* P*_cntP_*::*lacZ* (Zur-) | | KIM6+ Δ*lacZ* Δ*zur* P*_cntP_*::*lacZ*  *zur* deletion and *lacZ* reporter fusion with *cnt* promoter | |
| *Y. pestis ΔcntQ(RLMI) ΔybtX* | | KIM6+ Δ*cntQ*::Km1447 Δ*ybtX*::TpNP  *ybtX* deletion and insertion of a Tp resistance cassette without polar effect on downstream *ybt* genes | |
| *Y. pestis ΔcntLMI ΔznuCB* | | KIM6+ Δ*cntLMI*::Tp1087 Δ*znuCB*::Km4  Deletion of *znuCB* and insertion of a kanamycin resistance cassette | |
| *Y. pestis ΔcntQ(RLMI) ΔybtX ΔznuCB* | | KIM6+ Δ*cntQ*::Km1447 Δ*ybtX*::TpNP Δ*znuCB*::Sm1315  Deletion of *znuCB* and inserting a streptomycin resistance cassette | |
| **Plasmids** | | | |
| Name | Description | | Reference |
| pACYC177 | Suicide plasmid used as a template to generate pEP699 by ExoCET cloning. | | (Rose 1988) |
| pDS132 | Suicide plasmid, CmR. | | (Philippe et al. 2004) |
| pFLP3 | Encodes FLP recombinase to excise FRT flanked cassettes. | | (Choi et al. 2005) |
| pKD4 | Source of KmR cassette, Km flanked by FRT sites. | | (Datsenko and Wanner 2000) |
| pEP620 | lacZ reporter suicide plasmid bearing P*_cnt_* from *Y. pestis*, KmR | | This work |
| pEP681 | pDS132:: DcntQ::Km1447, CmR KmR | | This work |
| pEP692 | pDS132::DcntLMI::Tp1087, CmR TpR | | This work |
| pEP696 | lacZ reporter suicide plasmid bearing Pcnt from *Y. pseudotuberculosis*, derived from pEP848, KmR | | This work |
| pEP699 | p15A-Ap::cntPQRLMI, obtained by ExoCET cloning of the *Y. pseudotuberculosis cnt* operon, AmpR | | This work |
| pEP701 | pUC18R6K-miniTn7-Zeo2-cntPQRLMI, bears the *Y. pseudotuberculosis cnt* operon*,* AmpR ZeoR | | This work |
| pEP848 | lacZ reporter suicide plasmid, KmR | | EP collection |
| pEP1046 | pUC18R6K-miniTn7-Zeo2, used to clone the *Y. pseudotuberculosis cnt* operon,  AmpR ZeoR | | GenBank MH614591 |
| pEP1087 | Source of TpR cassette flanked by FRT sites | | (Bibi-Triki et al. 2014) |
| pEP1315 | Source of SmR cassette flanked by FRT sites | | GenBank MH626523 |
| pEP1436 | Plasmid used for Red recombination in *Y. pestis* | | (Dewitte et al. 2020) |
| pEP1447 | Source of Km1447 cassette flanked by FRT sites, in this plasmid the Km resistance gene is oriented opposite to that in pEP1446 | | EP collection |

**Supplementary Table 3. Composition of the TMH medium**

| **Compound** | **Final concentration (mM)** |
| --- | --- |
| DL-Ala | 2.5 |
| L-Ile | 1 |
| L-Val | 1 |
| L-Leu | 1 |
| L-Phe | 1 |
| L-Tyr | 1 |
| L-Thr | 5 |
| L-Arg | 1 |
| L-Pro | 5 |
| L-Glu | 5 |
| L-Lys | 1 |
| Gly | 5 |
| L-His | 1 |
| L-Asp | 2 |
| L-Ser | 5 |
| L-Trp | 0.1 |
| L-Asn | 2.5 |
| L-Gln | 10 |
| L-Met | 1 |
| Sodium thiosulfate | 2.5 |
| KH_2_PO_4_ | 2.5 |
| Citric acid | 10 |
| NH_4_Cl | 10 |
| MnCl_2_ | 0.01 |
| FeCl_2_ | 0.1 |
| MgCl_2_ | 20 |
| HEPES | 25 |
| Thiamine (vit. b2) | 0.0003 |
| Panthotenic acid (vit. b5) | 0.0004 |
| Biotine (vit. b8) | 0.0002 |
| Potassium gluconate | 10 |

**Supplementary Table 4. Metal concentrations in TMH medium.** Metal concentration as determined by ICP-AES (Bdl: below detection level).

| **Metal** | **Concentration (μM)** |
| --- | --- |
| Mn | 6.3 |
| Fe | 62.5 |
| Zn | Bdl |
| Cu | Bdl |
| Ni | Bdl |
| Co | Bdl |

**Supplementary References**

Bibi-Triki S, Li de la Sierra-Gallay I, Lazar N, et al (2014) Functional and Structural Analysis of HicA3-HicB3, a Novel Toxin-Antitoxin System of *Yersinia pestis*. Journal of Bacteriology 196:3712–3723. https://doi.org/10.1128/JB.01932-14

Choi K-H, Gaynor JB, White KG, et al (2005) A Tn7-based broad-range bacterial cloning and expression system. Nat Methods 2:443–448. https://doi.org/10.1038/nmeth765

Datsenko KA, Wanner BL (2000) One-step inactivation of chromosomal genes in *Escherichia coli* K-12 using PCR products. Proceedings of the National Academy of Sciences 97:6640–6645. https://doi.org/10.1073/pnas.120163297

Dewitte A, Bouvenot T, Pierre F, et al (2020) A refined model of how *Yersinia pestis* produces a transmissible infection in its flea vector. PLOS Pathogens 16:e1008440. https://doi.org/10.1371/journal.ppat.1008440

Philippe N, Alcaraz J-P, Coursange E, et al (2004) Improvement of pCVD442, a suicide plasmid for gene allele exchange in bacteria. Plasmid 51:246–255. https://doi.org/10.1016/j.plasmid.2004.02.003

Rose RE (1988) The nucleotide sequence of pACYC177. Nucleic Acids Research 16:356. https://doi.org/10.1093/nar/16.1.356
